# Interleukin-6 from Adipose-Derived Stem Cells Promotes Tissue Repair by the Increase of Cell Proliferation and Hair Follicles in Ischemia/Reperfusion-Treated Skin Flaps

**DOI:** 10.1155/2019/2343867

**Published:** 2019-11-13

**Authors:** Chi-Ming Pu, Ya-Chun Chen, Yu-Chen Chen, Tzu-Lin Lee, Yu-Sen Peng, Shun-Hua Chen, Yu-Hsiu Yen, Chung-Liang Chien, Jung-Hsien Hsieh, Yuh-Lien Chen

**Affiliations:** ^1^Department of Anatomy and Cell Biology, College of Medicine, National Taiwan University, Taipei, Taiwan; ^2^Division of Plastic Surgery, Department of Surgery, Cathay General Hospital, Taipei, Taiwan; ^3^Division of Nephrology, Department of Internal Medicine, Far-Eastern Memorial Hospital, New Taipei City, Taiwan; ^4^Department of Microbiology and Immunology, College of Medicine, National Cheng Kung University, Tainan, Taiwan; ^5^Division of Plastic Surgery, Department of Surgery, National Taiwan University Hospital, Taipei, Taiwan

## Abstract

The most common postoperative complication after reconstructive surgery is flap necrosis. Adipose-derived stem cells (ADSCs) and their secretomes are reported to mediate skin repair. This study was designed to investigate whether conditioned media from ADSCs (ADSC-CM) protects ischemia/reperfusion- (I/R-) induced injury in skin flaps by promoting cell proliferation and increasing the number of hair follicles. The mouse flap model of ischemia was ligating the long thoracic vessels for 3 h, followed by blood reperfusion. ADSC-CM was administered to the flaps, and their survival was observed on postoperative day 5. ADSC-CM treatment led to a significant increase in cell proliferation and the number of hair follicles. IL-6 levels in the lysate and CM from ADSCs were significantly higher than those from Hs68 fibroblasts. Furthermore, a strong decrease in cell proliferation and the number of hair follicles was observed after treatment with IL-6-neutralizing antibodies or si-IL-6-ADSC. In addition, ADSC transplantation increased flap repair, cell proliferation, and hair follicle number in I/R injury of IL-6-knockout mice. In conclusion, IL-6 secreted from ADSCs promotes the survival of I/R-induced flaps by increasing cell proliferation and the number of hair follicles. ADSCs represent a promising therapy for preventing skin flap necrosis following reconstructive and plastic surgery.

## 1. Introduction

Skin flap transplantation is frequently used in plastic and reconstructive surgery for its flexibility and convenience in repairing local tissue loss and its ability to correct tissue defects [1]. The trouble with skin flap transplantation for plastic surgeons is necrosis, which is the major complication following flap surgery. Total or partial flap failure may require additional reconstruction. Such complications increase the risk of injury site infections and postoperative hospitalizations, and they increase medical expenses [2, 3]. Current treatments include surgery, wound dressing, skin substitutes, and topical negative pressure; however, these methods are not sufficient for all circumstances, and there is an urgent demand to develop innovative therapies to reduce ischemia/reperfusion (I/R) injuries [4]. Stem cell-based therapies for I/R injury are a new field of medicine for regenerating tissues [5]. One adult stem cell candidate for regenerative medicine resides in the adipose tissue [6, 7]. Adipose-derived stem cells (ADSCs) are found in adipose stromal tissues and are multipotent stem cells that are capable of differentiating into multiple mesenchymal lineages [6]. Recently, ADSC transplantation was shown to induce angiogenesis in patients with critical limb ischemia and rats with acute kidney injuries, and it accelerated mouse excisional wound healing [8, 9]. Many factors are secreted by ADSCs, such as platelet-derived growth factor (PDGF), transforming growth factor-*β* (TGF-*β*), and vascular endothelial growth factor (VEGF). These factors are known to influence the repair of damaged tissues [10, 11]. However, no research has reported that the application of conditioned media from ADSCs could repair I/R-induced injury of skin flaps through an increase in cell proliferation and in hair follicle number.

The previous studies reported that proliferation of cells and skin appendages, including hair follicles, contributes to wound healing [12, 13]. Hair follicles have positive effects in wounds, and neofolliculogenesis is a natural process of normal regeneration versus fibrosis or chronic wounds [14]. An increase in the number of hair follicles in the graft indicates a better wound healing. At present, most clinical treatments for wound healing fail to achieve scarless skin regeneration with complete recovery of hair follicles. They lack many physiological functions of normal skin and seriously affect the quality of life of patients. However, functional regeneration of hair follicles in wound healing is a great challenge. Interleukin-6 (IL*-*6), a unique pleiotropic cytokine, has a wide range of biological activities in multiple systems [15]. It has been noted that the inflammatory response that occurs after cutaneous wounding is a major event for healing and that IL*-*6, an inflammatory cytokine, was involved in this process [16, 17]. The administration of a murine IL-6 expression plasmid or recombinant IL-6 reduced delayed wound healing in IL*-*6*-*deficient mice [18]. Our previous study demonstrated that IL-6 from ADSC-CM and ADSC-exosomes plays an important role in wound healing and angiogenesis after I/R injury of the skin flap [19]. However, further investigation is needed to elucidate the effects of the cytokines found in ADSC-CM on the production of hair follicles and the proliferation of cells. In the current study, we demonstrated that IL-6 in ADSC-CM increased cell proliferation and the number of hair follicles in the skin flap model for I/R injury via manipulation of the long thoracic artery. These findings showed that a cell-based therapy using endogenous stem cell populations from adipose tissues is an appropriate and innovative approach for treating I/R-induced injury.

## 2. Materials and Methods

### 2.1. Preparation of Human ADSCs

Human ADSC isolation was performed as previously described with some modifications [19]. Human abdominal subcutaneous adipose tissue was obtained after liposuction. This study was conducted after institutional ethical clearance, and permission was granted by the Human Ethics Committee of Cathay General Hospital (GGH-P103021). The isolated cells were cultured in flasks at 37°C with 5% CO_2_ in DMEM plus 20% fetal bovine serum (FBS) and antibiotics. The immunophenotypic characteristics (CD34, CD45, CD73, CD90, and CD105; all from BD Biosciences, CA, USA) of ADSCs were determined through flow cytometric analysis and immunocytochemistry. Adipogenic, osteogenic, and chondrogenic differentiations were used to identify the in vitro differentiation capacities of ADSCs.

### 2.2. Experimental Ischemia/Reperfusion Flap Model

The procedures for all animal studies were carried out in strict accordance with the guidelines for animal care of the National Taiwan University (No. 20150502) and complied with the Guide for the Care and Use of Laboratory Animals, NIH publication No. 86–23, revised 1985. Male C57BL/6J mice weighing 25 ± 5 g were housed under pathogen-free conditions in 12 : 12 light : dark cycles and had free access to standard chow and filtered water. C57BL/6J-derived IL6^−/−^ mice (B6.129S2-Il6tm1kopf/J, IL6 KO mice) were also used, and they were purchased from the Jackson Laboratory. Surgical procedures were carried out using standard aseptic conditions. Mice were anaesthetized by intraperitoneal injection of 50 mg/kg pentobarbital, and an extended pectoral skin flap (4 × 1 cm^2^) containing the right long thoracic vessels was revealed as reported in a previous study [19, 20]. The pedicle of the flap was then clamped to induce global ischemia. After 3 h, subsequent reperfusion was established by releasing the clamp. In the ADSC-CM group, the flap was sutured back into its native configuration, and ADSC-CM was applied to the subcutaneous layer between the flap and its bed. Injections were performed at the proximal, middle, and distal parts of the skin flap to confirm distribution across the entire flap. The I/R group received saline injections. Some mice did not undergo the ischemic operation; rather, the flap was raised and immediately sutured back, and these mice were the non-I/R sham group.

The animals were euthanized by lethal intravenous injection of 100 mg/kg pentobarbital on postoperative day 5. The harvested flaps were fixed in 10% buffered formalin and were then paraffin-embedded and sectioned. Paraffin sections (5 *μ*m thick) were dewaxed, rehydrated, and stained with hematoxylin and eosin. The histologic images were obtained from an Aperio CS2 digital pathology scanner. The number of hair follicles was viewed under high-power fields. All specimens were assessed separately by two dermatologists who were blinded to the different groups.

### 2.3. Western Blot Analysis

Western blot analyses of proteins in cell lysates and conditioned media were performed based on a methodology that was used in our previous study [21]. In brief, samples were run on 10% polyacrylamide electrophoresis gels and then were transferred to nitrocellulose membranes. Subsequently, the membranes were blocked with 5% BSA (Sigma, MA, USA) in Tris-buffered saline for 1 h. Then, these membranes were incubated with primary antibodies to IL-6 (1 : 1000, Abcam, MA, USA) or GAPDH (1 : 1000, Santa Cruz, TX, USA) in TBS-1% Tween containing 5% BSA at 4°C. After incubation, the cells were probed for 1 h with the appropriate HRP-conjugated secondary Ab (1 : 5000, GeneTex, CA, USA). The bound antibodies were visualized by chemiluminescence using a BioSpectrum 600 imaging system (UVP, CA, USA).

### 2.4. Immunofluorescent Staining

To determine the difference in IL-6 expression between ADSCs and fibroblasts (Hs68 human forskin fibroblasts, ATCC), the cells were incubated overnight at 4°C with rabbit polyclonal anti-IL-6 antibody (Abcam) diluted 1 : 500. Then, the cells were incubated with FITC anti-rabbit IgG (1 : 200 dilution, Vector Laboratories, CA, USA) for 1 h at room temperature. The result was viewed by fluorescence microscopy.

### 2.5. siRNA Transduction and IL-6 Neutralization Experiment

Accell SMARTpool siRNA (Dharmacon, Inc., PA, USA) targeted IL-6 to silence it. A 100 *μ*M stock of IL-6 siRNA was prepared in RNase-free water and stored at -20°C. The nontarget Accell siRNA comprising a scrambled sequence was the control, and it had no significant homology to human gene sequences; it was stored in Accell siRNA delivery media (Dharmacon, Inc.). ADSCs were seeded in a 6-well plate (Sarstedt) at 70-80% confluence and incubated for 24 h. Subsequently, the culture media were replaced with a solution containing 1 *μ*M IL-6 siRNA or control siRNA. Cells were then cultured in a 5% CO_2_ incubator at 37°С for 72 h [22]. The downregulation of IL-6 was confirmed by ELISA. For the IL-6 neutralization experiment, 5 *μ*g/mL of IL-6-neutralizing antibody (BioLegend, CA, USA) or immunoglobulin (IgG, BioLegend) was added to the conditioned medium. The conditioned media collected from ADSCs treated with IL-6 siRNA, or with scrambled siRNA, or with IL-6-neutralizing antibody, or with immunoglobulin, which are referred to as the si-IL-6 and si-Scramble, anti-IL6, and IgG groups, respectively, were used to study the role of IL-6 in the I/R-induced flap injury animal study.

### 2.6. Immunostaining

To identify the expression of proliferating cell nuclear antigen (PCNA), sections from different treatments were stained using rabbit polyclonal anti-PCNA antibody (Santa Cruz) diluted 1 : 500. We used a streptavidin-biotin-peroxidase technique (rabbit-specific HRP detection kit) according to the manufacturer's recommendations (Abcam). The color was enhanced using a 3,3′-diaminobenzidine tetrahydrochloride substrate kit (Abcam). The sections were counterstained with hematoxylin. The proportion of stained cells in each sample was calculated.

### 2.7. Statistical Analysis

The data are shown as the means ± SEM for three to six separate experiments and are expressed as a fold value compared to the control value unless other specified. The significant differences in the means of the data were examined using a one-way ANOVA and a Fisher's test. Values were considered significant when *p* < 0.05.

## 3. Results

### 3.1. Treatment with ADSC-CM Increased Cell Proliferation and the Number of Hair Follicle in I/R-Induced Flaps

**Figure 1:**
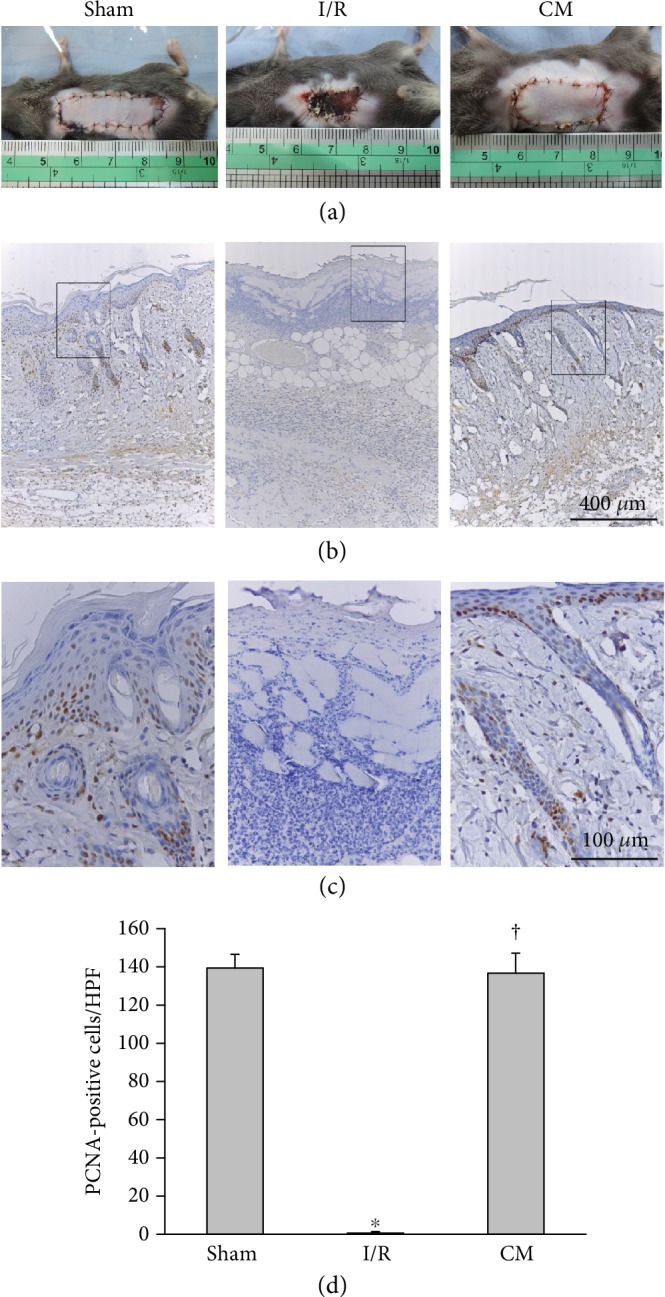
ADSC-CM transplantation enhanced cell proliferation after I/R operation. (a) Flaps (4 × 1 cm^2^) of mice with ischemia induced by ligating long thoracic vessels for 3 h, which was then followed by blood reperfusion. ADSC-CM was administered into flaps through a subcutaneous route. Representative photographs of skin flaps on postoperative day 5 are shown. The necrotic areas of the I/R-induced skin flap were much larger than those of the sham group. In contrast, ADSC-CM (CM) treatment reduced the necrotic areas induced by I/R injury. (b) Immunostaining of PCNA. Bar = 400 *μ*m. (c) Higher magnification of the area inside the rectangle in (b) is displayed. The PCNA-positive cells localized to the basal layer of the epidermis and the hair follicles. Bar = 100 *μ*m. (d) Statistical analysis of PCNA-positive cells under high-power field (HPF) using ImagePro software. *n* = 6 for each group. ^∗^*p* < 0.05 versus the sham group; ^†^*p* < 0.05 versus the I/R group.

**Figure 2:**
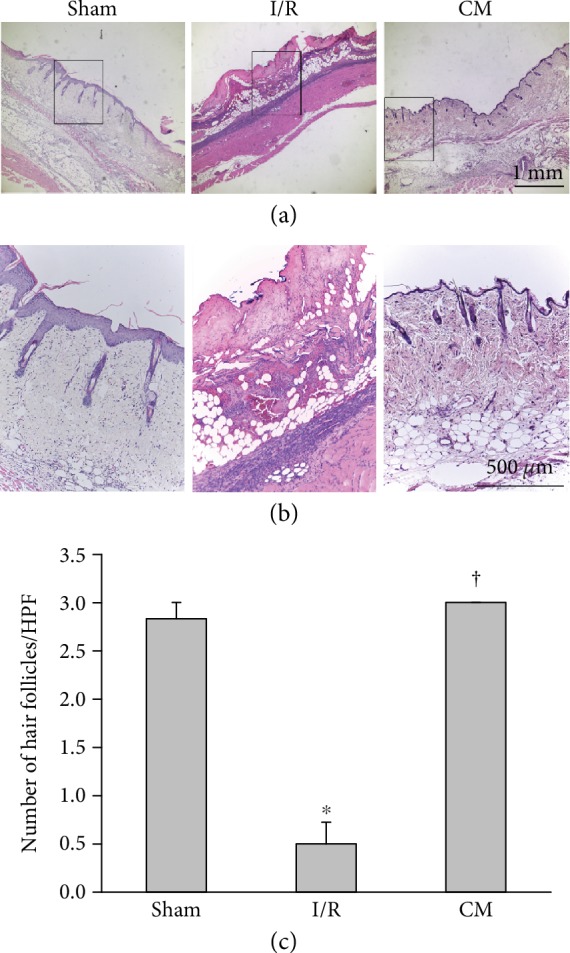
ADSC-CM transplantation increased the number of hair follicles after I/R operation. (a) Sections were stained with hematoxylin and eosin. Bar = 1 mm. (b) Higher magnification of the area inside the rectangle in (a) is shown. Bar = 500 *μ*m. (c) Statistical analysis of hair follicles under HPF. *n* = 6 for each group. ^∗^*p* < 0.05 versus the sham group; ^†^*p* < 0.05 versus the I/R group.

The characteristics of ADSCs in this study were similar to those reported in our previous study [19]. A pectoral skin flap was created to investigate whether cell proliferation played a crucial role in the recovery of I/R-induced injury to the skin flap. The necrosis of the skin flap was clearly observed in the I/R mice, whereas the ADSC-CM treatment attenuated the I/R-induced necrotic area (Figure 1(a)). Cell proliferation was reduced in the I/R group compared with the sham group, as shown by PCNA immunostaining (Figure 1(b)). In contrast, ADSC-CM treatment reversed the detrimental proliferation effect induced by I/R. The marked rectangular area is shown at higher magnification in Figure 1(c). PCNA-positive cells were abundant in the basal layer of the epidermis and epithelium of hair follicular bulbs. Quantitative analysis of the number of proliferative cells among the three groups is shown in Figure 1(d). We further used hematoxylin and eosin staining to examine the effects of ADSC-CM on the number of hair follicles in I/R-induced flaps. The I/R+ADSC-CM group showed many hair follicles when compared with the I/R group (Figure 2(a) and 2(b)). Quantitative analysis of the number of hair follicles in the I/R group was significantly attenuated, whereas ADSC-CM treatment increased the number (Figure 2(c)).

### 3.2. IL-6 from ADSCs Promoted Cell Proliferation and Increased the Number of Hair Follicles

**Figure 3:**
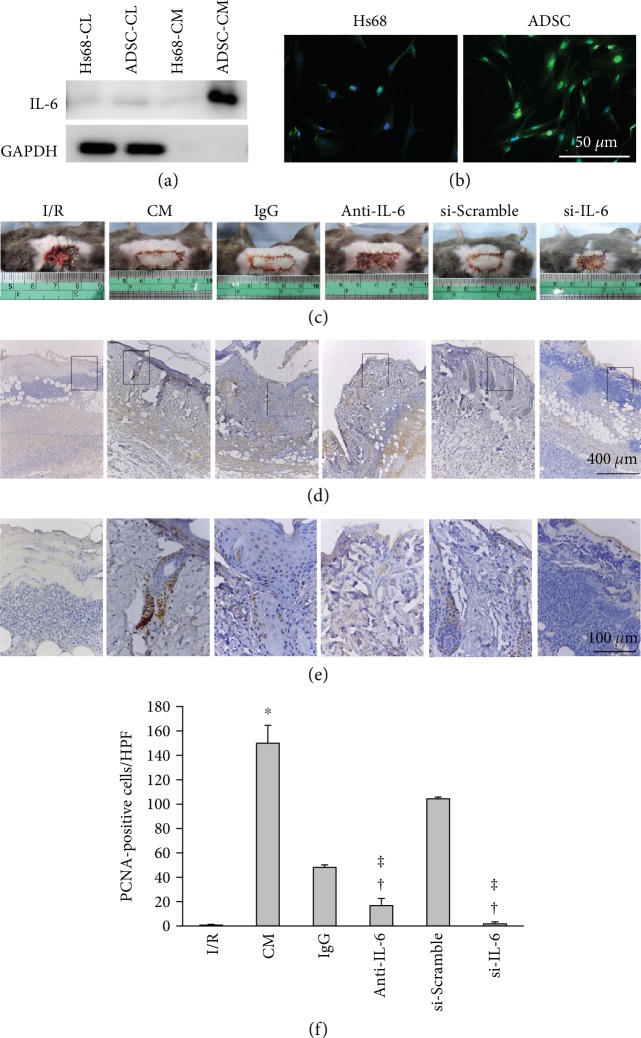
IL-6 in ADSC-CM promoted flap recovery through an increase of cell proliferation. (a) Levels of IL-6 expression in the cell lysates (CL) or in the conditioned medium (CM) from ADSCs and Hs68 fibroblasts were examined by Western blot. (b) The intensity of IL-6 expression in ADSCs was stronger than it was in Hs68 cells, as shown by immunocytochemical staining. Bar = 50 *μ*m. (c) The conditioned media were collected from ADSCs pretreated with or without a control immunoglobulin G (IgG), an IL-6-neutralizing antibody (anti-IL-6), an IL-6 siRNA transfection (si-IL-6), or a control scrambled oligonucleotide sequence (si-Scramble, si-Con) transfection. Representative images of the I/R-induced skin flaps in the presence or absence of CM, IgG, anti-IL-6, si-Con, or si-IL-6 treatment on postoperative day 5. (d) Photographs of I/R-induced skin flaps in the presence or absence of ADSC-CM, ADSC+IgG-CM (IgG), ADSC+anti-IL-6 antibody-CM (anti-IL-6), si-Scramble-ADSC-CM (si-Con), or si-IL-6-ADSC-CM (si-IL-6). The sections were stained with PCNA antibodies. Bar = 400 *μ*m. (e) Higher magnification of the area enclosed by the rectangle in (d) is shown. Bar = 100 *μ*m. (f) Statistical analysis of PCNA-positive cells under HPF. *n* = 6 for each group. ^∗^*p* < 0.05 versus the I/R group; ^†^*p* < 0.05 versus the CM group; ^ǂ^*p* < 0.05 versus the IgG or the si-Scramble group.

Our previous study demonstrated that treatment with both ADSC and IL-6 secreted from ADSCs could effectively enhance skin flap recovery and stimulate angiogenesis after I/R injury [19]. Here, we investigated whether ADSC-derived IL-6 reverses I/R-induced injury by enhancing cell proliferation and increasing the number of hair follicles in skin flaps. IL-6 expression was higher in cell lysates and conditioned media from ADSCs than in the lysate and media from Hs68 cells (these cells represent a major component of dermis), as determined by Western blot (Figure 3(a)). Immunofluorescent staining was also consistent with this result (Figure 3(b)). Furthermore, to explore whether IL-6 was involved in cell proliferation, I/R flaps were treated with different conditioned media as follows. Conditioned media were collected from cultured ADSCs with different treatments of a control immunoglobulin G (IgG), IL-6-neutralizing antibody (anti-IL-6), IL-6 siRNA transfection (si-IL-6), or a control scrambled oligonucleotide sequence transfection (si-Scramble). ELISA assay was used to examine the efficiency of IL-6 knockdown. IL-6 levels in ADSC-CM (122.7 ± 4.2 pg/mL) were significantly higher than those from the si-IL-6 group (30.4 ± 1.2 pg/mL). IL-6 levels in the si-Scramble group were 94.0 ± 6.8 pg/mL. The I/R group, the anti-IL-6 antibody group, and the IL-6-silencing group had severe necrotic areas, whereas the IgG group and the si-Scramble group exhibited better recovery of I/R-induced injury (Figure 3(c)). The anti-IL-6 antibody group and the IL-6-silencing group showed a smaller number of proliferative cells compared to the IgG group and the si-Scramble group, respectively (Figure 3(d) and 3(e)). The groups treated with anti-IL-6 antibody or with IL-6 silencing had significantly attenuated cell proliferation compared with the IgG and si-Scramble groups, respectively (Figure 3(f)).

**Figure 4:**
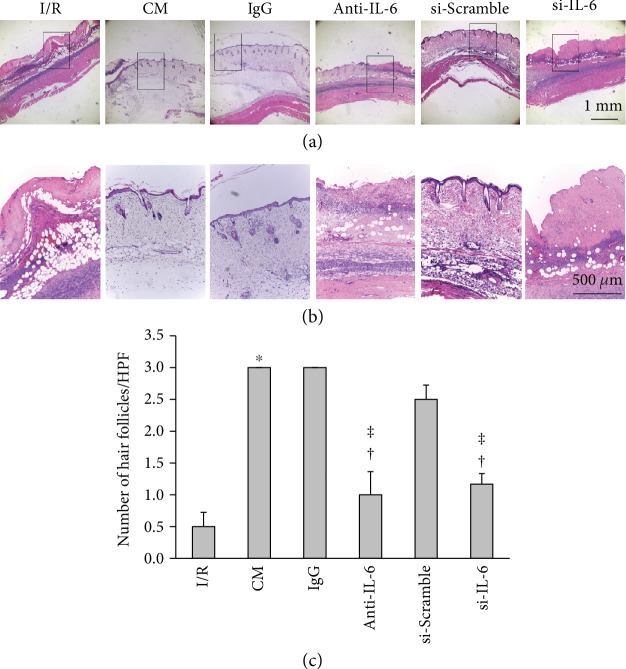
IL-6 in ADSC-CM promoted flap recovery and increased the number of hair follicles. (a) The conditioned media were collected from ADSCs pretreated with or without a control immunoglobulin G (IgG), an IL-6-neutralizing antibody (anti-IL-6), an IL-6 siRNA transfection (si-IL-6), or a control scrambled oligonucleotide sequence (si-Scramble, si-Con) transfection. Photographs of I/R-induced flaps in the presence or absence of ADSC-CM, ADSC+IgG-CM (IgG), ADSC+anti-IL-6 antibody-CM (anti-IL-6), si-Scramble-ADSC-CM (si-Scramble), or si-IL-6-ADSC-CM (si-IL-6). The sections were stained with H&E. Bar = 1 mm. (b) Higher magnification of the area enclosed by the rectangles in (a). Bar = 500 *μ*m. (c) Statistical analysis of hair follicles under HPF. *n* = 6 for each group. ^∗^*p* < 0.05 versus the I/R group; ^†^*p* < 0.05 versus the CM group; ^ǂ^*p* < 0.05 versus the IgG or the si-Scramble group.

A previous study demonstrated that IL-6 is important for the growth of hair follicles [23]. We therefore tested whether the effect of ADSC-CM on the number of hair follicles was attributable to IL-6. Both the anti-IL-6 antibody and the IL-6-silencing groups had significantly reduced flap repair and had significantly fewer hair follicles than the IgG and si-Scramble groups, respectively (Figure 4(a) and 4(b)). The number of hair follicles in the anti-IL-6 antibody and IL-6-silencing groups showed a significantly reduced number of hair follicles, respectively, compared to the IgG or the si-Scramble group (Figure 4(c)). Taken together, these results demonstrate that ADSC-CM promoted flap recovery, increased cell proliferation, and increased the induction of hair follicles through IL-6 signaling.

### 3.3. IL-6 in ADSCs Enhanced the Production of Cell Proliferation and Hair Follicles in IL-6 KO Mice

**Figure 5:**
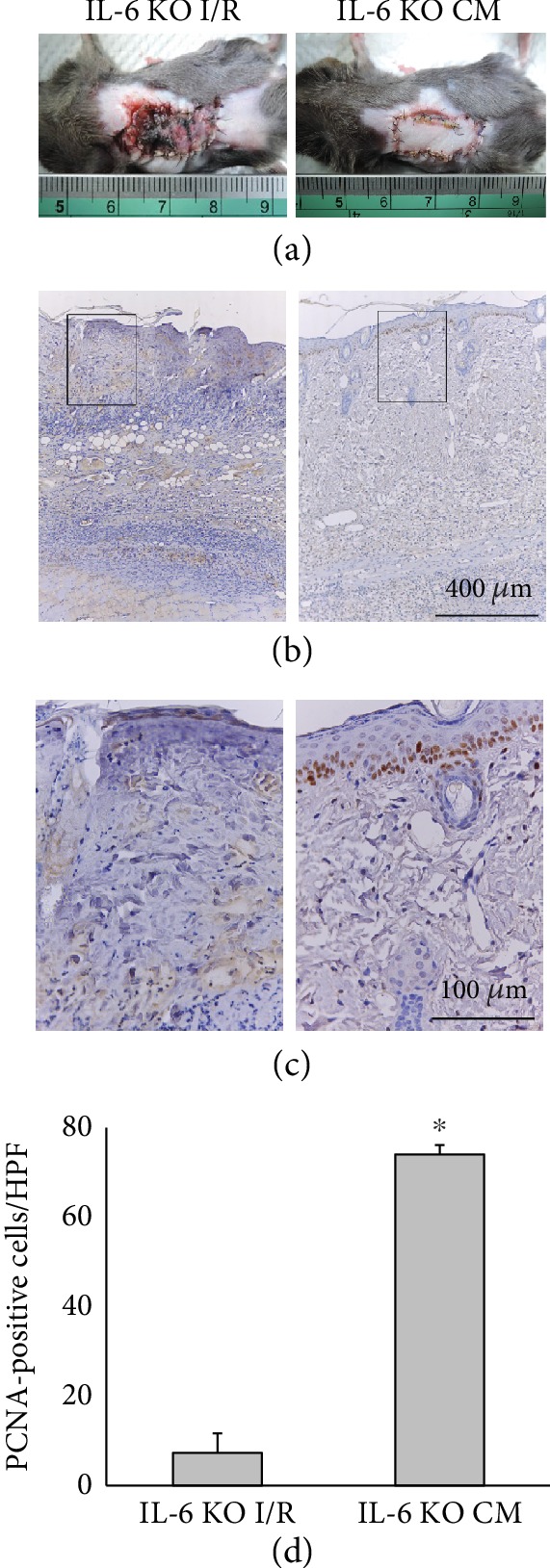
IL-6 in ADSCs promoted flap recovery through the increased cell proliferation in IL-6 KO mice. (a) Representative photographs of skin flaps with or without ADSC treatment on postoperative day 5 of I/R in IL-6 KO mice. (b) Flap sections were immunostained with anti-PCNA antibody. Bar = 400 *μ*m. (c) The rectangles in (b) are magnified and shown in (c). Bar = 100 *μ*m. (d) Histogram showing the number of proliferating cell under HPF. ^∗^*p* < 0.05 versus the IL-6 KO I/R group.

IL-6 KO mice were utilized in our study to directly address the role of IL-6 in cell proliferation and hair follicle induction in an I/R injury model. We found that IL-6 KO mice suffered severe skin flap necrosis, while ADSC administration ameliorated the I/R-induced injury (Figure 5(a)). Furthermore, the I/R-induced skin flaps with ADSC treatment had an increased number of proliferating cells in IL-6 KO mice, as shown by immunostaining for PCNA (Figure 5(b) and 5(c)). Quantitative analysis of the number of proliferating cells among the two groups is shown in Figure 5(d).

**Figure 6:**
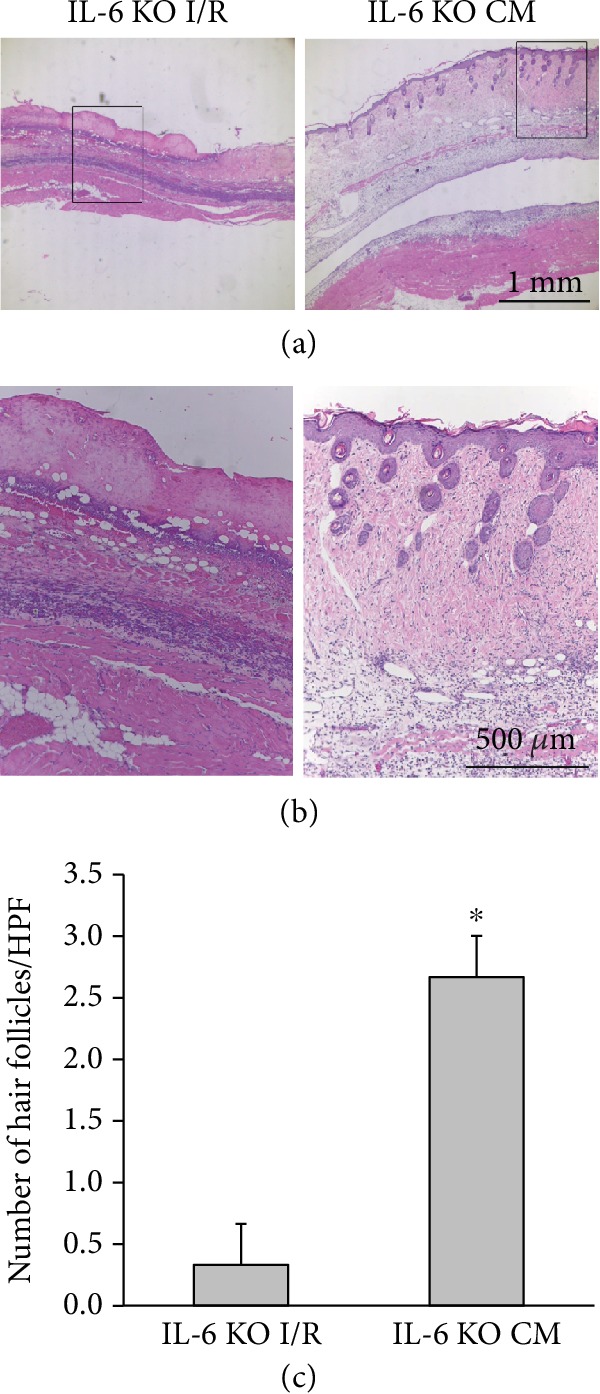
IL-6 in ADSCs promoted flap recovery through the increased number of hair follicles in IL-6 KO mice. (a) Flap sections with or without ADSC treatment at postoperative day 5 of I/R in IL-6 KO mice were stained with hematoxylin and eosin. Bar = 1 mm. (b) The rectangle boxes in (a) are magnified and shown in (b). Bar = 500 *μ*m. (c) Statistical analysis of the number of hair follicles under HPF. ^∗^*p* < 0.05 versus the IL-6 KO I/R group.

In addition, IL-6 KO mice showed few hair follicles in the I/R-induced skin flap, as shown by H&E staining, whereas the administration of ADSCs led to remarkably abundant hair follicles (Figure 6(a) and 6(b)). The score of hair follicles in the ADSC group was significantly higher than that of the I/R group (Figure 6(c)). Overall, these results indicate that IL-6 signaling is crucial for cell proliferation and hair follicle induction during I/R injury.

## 4. Discussion

The main findings in this study were that ADSC-CM significantly increased the skin survival, the amount of cell proliferation, and the number of hair follicles in I/R-induced skin flaps. These effects were mediated through IL-6. These results suggest that ADSCs may serve as a promising option for cell- and cytokine-based therapies of I/R-induced injury.

ADSCs were easily obtained from liposuction aspirates and easily grown in vitro. ADSCs have the potential to differentiate into a variety of mesodermal lineages [24] and are less vulnerable to immunological reactions [7, 25]. Because of the benefits, ADSCs are superior to stem cells derived from other sources [25]. Recently, ADSCs have been widely used across many clinical fields, especially for skincare and treating dermal wounds. For instance, ADSCs accelerated the process of wound closure in diabetic mice [26]. ADSCs injected at the flap pedicle improved the viability of random pattern skin flaps [27]. ADSCs prevented I/R injury using in extended inferior epigastric skin flaps [28]. In addition, ADSC-CM accelerated healing in 3-dimensional skin cultures [29]. Our previous report showed that ADSC protected pectoral skin flaps after I/R injury [19]. Consistent with the previous report, we showed that the I/R-induced skin flap treated with local injections of ADSC-CM exhibited enhanced flap repair. Furthermore, we showed that I/R injuries reduced the cell proliferation and the number of hair follicles compared to the control group, while ADSC-CM treatment revered these effects.

Cell proliferation is an essential process for wound healing [13]. Treatment with extract from adipose tissue significantly induced proliferation of cultured keratinocytes compared to plasma and control treatments [30]. Treatment with orbital adipose-derived stem cell-CM enabled the maintenance of polygonal cell morphology and the enhancement of proliferative capacity [31]. Importantly, the present study demonstrated that ADSC-CM induced cell proliferation in I/R-induced skin flaps, as shown by PCNA immunohistochemistry. PCNA was strongly expressed in the basal layer of the epidermis and in the epithelium of hair follicular bulbs. These results suggested that ADSCs promoted skin flap survival by augmenting cell proliferation. In addition, these studies reported that skin appendages, including hair follicles, played an important role in wound healing [12, 14]. In a process that used a composite acellular amniotic membrane and adipose-derived mesenchymal stem cells, hair follicle development was observed, and it repaired full-thickness skin defects [32]. Sericin hydrogels promote skin wound healing with effective regeneration of hair follicles and sebaceous glands after complete loss of epidermis and dermis [33]. The subcutaneous injection of enriched adipose tissue into the scalp of patients with early genetic alopecia enhanced their hair follicle growth [34]. A previous study also reported that ADSC-CM promoted hair growth and that this effect may be mediated by a paracrine mechanism [35]. We demonstrated that I/R significantly reduced the number of hair follicles and that ADSC-CM treatment remarkably increased the number. The results suggested that ADSC-CM treatment achieved functional recovery of the wounded skin.

Stem cells exhibit the ability to alter the tissue microenvironment through secretion of cytokines and can therefore contribute to wound healing [36]. Our previous study showed by antibody array blot that IL-6 was the most significantly changed angiogenic factor in ADSC-CM [19]. The present study demonstrated higher levels of IL-6 in ADSCs than in fibroblasts. Notably, ADSC-CM was found to have a significantly high level of IL-6. Previous studies have reported that IL-6, a pleiotropic cytokine, is related to the regulation of inflammation and angiogenesis [15, 37]. IL-6 KO mice subjected to brain ischemia exhibited an impaired angiogenic response with a reduced number of small vessels [38]. IL-6 derived from endothelial cells induces angiogenesis [39]. In addition, ADSC-derived IL-6 increased the proliferation of cardiomyocytes [40]. The present study demonstrated that conditioned media from ADSCs treated with an IL-6-neutralizing antibody or with IL-6 silencing showed decreased levels of tissue repair as well as decreased cell proliferation and fewer hair follicles. We also showed that IL-6 KO mice exhibited impaired recovery with a low number of proliferative cells and few hair follicles in response to I/R injury of the skin flap. ADSC treatment reversed these effects. Our data strongly suggested that IL-6 from ADSCs functionally promoted the number of proliferative cells and the number of hair follicles and then influenced tissue repair.

In summary, our study demonstrates that IL-6 secreted into ADSC-CM can effectively increase the survival of skin flaps as well as the number of hair follicles and the amount of cell proliferation following I/R injury. This mechanism of enhanced flap survival might occur because of the ability of ADSCs to produce IL-6. Although further studies are required to translate our experimental results into a broad clinical application, we believe that the function of ADSC and ADSC-CM demonstrated above represents a promising strategy for preventing I/R-induced necrosis of skin flaps following reconstructive and plastic surgery.

## Data Availability

The data used to support the findings of this study are included within the article.
